# Herpes Simplex Virus type 1 inhibits autophagy in glial cells but requires ATG5 for the success of viral replication

**DOI:** 10.3389/fmicb.2024.1411655

**Published:** 2024-06-10

**Authors:** Inés Ripa, Sabina Andreu, Fernando Josa-Prado, Beatriz Fernández Gómez, Fernando de Castro, María Arribas, Raquel Bello-Morales, José Antonio López-Guerrero

**Affiliations:** ^1^Department of Molecular Biology, Universidad Autónoma de Madrid, Madrid, Spain; ^2^Centro de Biología Molecular Severo Ochoa, CSIC-UAM, Madrid, Spain; ^3^Grupo de Neurobiología del Desarrollo-GNDe, Instituto Cajal-CSIC, Madrid, Spain

**Keywords:** Herpes Simplex Virus type 1, autophagy, multiple sclerosis, glial cells, oligodendrocyte, ATG5

## Abstract

Herpes Simplex Virus type 1 (HSV-1) 1 is a neurotropic virus that has been associated with neurodegenerative disorders. The dysregulation of autophagy by HSV-1 has been proposed as a potential cause of neurodegeneration. While studies have extensively tackled the interaction between autophagy and HSV-1 in neurons, research in glial cells is currently limited. Our studies demonstrate that HSV-1 inhibits, but not completely blocks, the formation of autophagosomes in human oligodendroglioma- and astrocytoma- derived cell lines. These findings have been confirmed in murine oligodendrocyte precursor cells (OPCs). Finally, this study investigates the impact of autophagy on HSV-1 infection in glial cells. While the lack of basal autophagy in LC3B knockout glial cells does not have a significant effect on viral infection, cells without the autophagy-related protein ATG5 exhibit reduced viral production. The absence of ATG5 leads to a decrease in the transcription and replication of viral genes, as well as a delay in the initial stages of the formation of HSV-1 replication compartments. These findings indicate that while autophagy may not play a significant role in antiviral defense in glial cells, HSV-1 may be inhibiting autophagy to exploit non-canonical functions of certain components of the autophagic machinery, such as ATG5, to benefit its lifecycle.

## 1 Introduction

Autophagy is a catabolic process by which defective proteins, complexes, and organelles are degraded, preventing the accumulation of potentially cytotoxic damage, and maintaining the cellular homeostasis (Ohsumi, 2014). Maintaining a well-balanced autophagy is critical for the proper development, functionality, and viability of the Central Nervous System (CNS) (Misrielal et al., 2020). Autophagy in the CNS has been studied predominantly in neurons, with limited exploration in glial cells, despite these cells constitute a large fraction of the mammalian brain (Herculano-Houzel, 2014) and play crucial roles in homeostasis and pathogenesis of the CNS. A growing interest in glial autophagy is emerging due to the regulation of this process appears to exhibit distinctions between neuronal and non-neuronal cell types (Belgrad et al., 2020).

Impaired autophagy in glial cells can disrupt their supportive functions, ultimately contributing to the pathogenesis of various neurological disorders, such as Multiple Sclerosis (MS) (Nutma et al., 2021). Examining autophagy in the context of MS is particularly relevant since this neurological condition is characterized by inflammation, demyelination, and neurodegeneration, all of which have been associated with autophagy (Hassanpour et al., 2020). A notable decrease in OLs has been observed within MS lesions (Wolswijk, 2002). OLs are myelinating cells derived from oligodendrocyte progenitor cells (OPCs) that hold a critical role in myelinating axons in the CNS (Fang and Bai, 2023). Autophagy assumes a significant role in promoting the survival and differentiation of OLs, thereby influencing their capacity to create myelin sheaths. Dysfunction of autophagy in OLs may be involved in cytoplasm decompaction and decreased numbers of myelin wraps, that ultimately leads to demyelination in MS (Hill et al., 2018). Another significant cell type in the context of MS is astrocytes, which play crucial roles in supporting neuronal communication, maintaining the integrity of the blood–brain barrier, and contributing to CNS lesion repair (Siracusa et al., 2019). Autophagy in astrocytes is involved in regulating mitochondrial dynamics and network organization during inflammation. Consequently, any impairment in this process results in the generation of reactive oxygen species (ROS), which amplifies the proinflammatory response and leads to astrocyte cell death (Lee et al., 2009; Motori et al., 2013).

Although the etiology of MS remains elusive, it is believed that the susceptibility to the disease is a combination of genetic and environmental factors, with viral infections being one of the environmental factors under scrutiny. Certain viruses of the Herpesviridae family have been linked to MS (Sedighi et al., 2023), with Herpes Simplex Virus type 1 (HSV-1) being among them (Bello-Morales et al., 2020). HSV-1 is a neurotropic virus that, after a primary infection of epithelial cells, traffics from the axon terminal to the trigeminal ganglia (Miranda-Saksena et al., 2018), where it establishes a lifelong latency (Singh and Tscharke, 2020). Occasionally, HSV-1 can spread to the CNS and initiate either a quiescent latent state (Yao et al., 2014) or a severe acute encephalitis (Mancini and Vidal, 2018). Indeed, HSV-1 infection of the CNS has been linked to neurodegenerative disorders (Duarte et al., 2019), such as Alzheimer’s disease (Mancuso et al., 2019), and to the onset and exacerbation of MS disease (Duarte et al., 2023). The link between HSV-1 and MS has primarily relied on indirect evidence, including the identification of viral genetic material in tissue samples or in body fluids of MS patients. Further evidence came from *in vivo* studies, in which HSV-1 infection of murine models have been proposed to cause demyelinating lesions (Jakhmola et al., 2021).

In the context of HSV-1 infection in the CNS, autophagy emerges as a potent host defense mechanism that serves to constrain HSV-1 pathogenesis within neurons (Yordy et al., 2012). However, HSV-1 has developed several strategies to hijack and block some steps of autophagy, thereby evading the immune response and promoting viral replication (Ripa et al., 2022). The modulation of autophagy by HSV-1 can alter the cellular homeostasis. For example, the inhibition of autophagy by HSV-1 in neurons has been linked to the accumulation of aggregates seen in Alzheimer’s disease (Santana et al., 2012a,b).

Despite autophagy is a high conserved process and the components of the autophagy machinery are widely expressed, the modulation of this process can exhibit organ-specific characteristics (Mizushima et al., 2004) and variation in different brain regions (Nikoletopoulou et al., 2017) and neuronal subtypes (Stavoe et al., 2016). Therefore, it is not surprising that autophagy exhibits distinct regulation in neurons as compared to glial cells (Pamenter et al., 2012; Moruno-Manchon et al., 2018). While the association between autophagy and HSV-1 infection in neurons is well-documented, our understanding of this interaction in glial cells remains limited. In the present study, we analyze the modulation of autophagy by HSV-1 in both glial cell lines and murine cultures of OPCs. On the other hand, we investigate the impact of autophagy on HSV-1- infection of glial cells.

One of the most common methodologies employed to analyze autophagy in herpesvirus infection is the silencing of the autophagy-related gene ATG5, due to the ATG5 protein is essential for autophagosome formation (Yakoub and Shukla, 2015a). Given its involvement in autophagy, ATG5 has traditionally been considered a protein with antiviral properties in HSV-1 infection (Tallóczy et al., 2006). However, recent research indicates that the role of this protein in viral infections is more complex than previously thought, and the participation of ATG5 in non-autophagic processes is attracting increasing interest. In this paper, we further investigate the non-canonical role of ATG5 in HSV-1 infection in glial cells.

## 2 Materials and methods

Cell lines. The U-87 MG cell line, kindly provided by María Jesús Bullido (CBMSO, Madrid, Spain), was cultured in Minimum Essential Medium (MEM) supplemented with 10% (vol/vol) fetal bovine serum (FBS), 2 mM glutamine, penicillin (50 U/mL) and streptomycin (50 μg/mL). The HOG cell line, provided by A. T. Campagnoni (University of California, Los Angeles, United States) and the Vero cell line, provided by Dr. Enrique Tabarés (UAM, Madrid, Spain), were cultured in low-glucose Dulbecco’s Modified Eagle’s Medium (DMEM) containing 5% FBS, glutamine and antibiotics. Cells were incubated at 37°C in an atmosphere of 5% CO_2_. All the experiments were performed with HOG cells previously cultured in differentiation medium (DM) for 24 h, as previously described (Bello-Morales et al., 2014).

Isolation and culture of oligodendrocyte precursor cells (OPCs). Extraction and culture of OPCs were performed as previously reported (Dincman et al., 2012), with substantial modifications (Bribián et al., 2020; Melero-Jerez et al., 2021). CD-1 mice (6–7 days old) were provided by the animal facility of the Centro de Biología Molecular Severo Ochoa-CBMSO (CSIC-UAM, Madrid, Spain). All animal procedures were performed in compliance with the European guidelines for Animal Research (European Communities Council Directives 2010/63/EU, 90/219/EEC, Regulation (EC) No.1946/2003), and were approved by the Ethical Review Board of Consejo Superior de Investigaciones Biológicas-CSIC and Comunidad de Madrid.

Mouse brains were removed (this protocol is suitable for working with 2–3 brains) and immersed in pre-chilled HBSS containing Ca^+^ and Mg^+^ (Gibco). The cerebellum, brainstem, olfactory bulbs, and olfactory tracts were then removed, followed by the dissection of the meninges from the remaining cortex. The dissected cortices were transferred to pre-chilled HBSS without Ca^+^ and Mg^+^ (Gibco). The cortices were diced with a razor blade, then 10 mL of HBSS was added and the tissue was centrifuged at 300 × g for 2 min at room temperature (RT), the supernatant being discarded. The subsequent disaggregation of the tissue was performed using the Neural Tissue Dissociation Kit (Miltenyi Biotec, 130-092-628). After disaggregation of the tissue, the suspension was decanted through a 70 μm strainer (Merck). The tissue was then centrifuged at 300 × g for 10 min at RT and the supernatant was completely removed. The cell pellet was resuspended in anti-O4 microbeads (Miltenyi Biotec, 130-096-670) diluted in MACS buffer (10% v/v). The MACS buffer consists of 0.5% bovine serum albumin (BSA) (Sigma), 2 mM sodium pyruvate (Gibco) and 2 mM EDTA (Invitrogen) in PBS 1X. Cells were incubated with the microbeads for 30 min at 4°C with gentle shaking. The cell suspension was then washed by adding 2 mL of MACS buffer followed by centrifugation at 300 × g for 10 min. During centrifugation, a MACS MS column (Miltenyi Biotec, 130-042-201) was placed on a MACS magnetic stand (Miltenyi Biotec, 130-042-102) and the column was equilibrated with 0.5 mL of MACS buffer. Finally, the pellet was resuspended in 0.5 mL MACS buffer and the cell suspension was decanted through a 40 μm strainer. The cell suspension which passed through the filter was added to the column and washed 3 times with MACS buffer. The column was then removed from the magnet, and the bound cells were eluted in 0.5 mL of pre-heated OPC growth medium. The growth medium for OPCs is based on MACS neuromedium (Miltenyi Biotec, 130-093-263) supplemented with 2 mM glutamine, penicillin (50 U/mL), streptomycin (50 μg/mL), 0.5 M glucose, 2% MACS neurobrew-21 (Miltenyi Biotec, 130-097-263), 10 ng/mL human fibroblast growth factor (FGF) (Peprotech, 100-18B) and 10 ng/mL human platelet-derived growth factor-AA (PDGF-AA) (Peprotech, 100-13A). Cells were counted and plated in a poly-L-lysine (PLL)/laminin-coated 24-well plate. Laminin was purchased from Sigma (L2020) and PLL solution was purchased from Santa Cruz Animal Health (CAS-25988-63-0). Cells were incubated at 37°C, 5% CO_2_, and the day after OPC isolation, the medium was removed from the plate and replaced with fresh medium. For OPC differentiation, cells were cultured for 3 days with fresh OPC medium without growth factors.

Generation of ATG5 and LC3B knockout cell lines. Alt-R CRISPR-Cas9 crRNAs (Table 1) were mixed with Alt-R CRISPR-Cas9 tracrRNA and heated at 95°C for 5 min. crRNA:tracrRNA duplexes were incubated with Alt-R S.p. HiFi Cas9 Nuclease V3 to assemble the ribonucleoprotein (RNP) complex. For RNP complex transfection, HOG and U-87 MG cells were electroporated using the Alt-R Cas9 Electroporation Enhancer. Alt-R CRISPR-Cas9 system components were purchased from Integrated DNA Technologies (IDT). To grow single cell clones, transfected cells were cultured in a 96-well plate at a density of 0.5 cells/well. Finally, the autophagy deficiency of KO cells was tested by immunoblotting. Primer design and RNP transfection were performed by the Transgenesis Core Facility at the Centro Nacional de Biotecnología-CNB (CSIC, Madrid, Spain).

**Table 1:** crRNAs for knockout the human *ATG5* and *MAP1LC3B* genes by the CRISPR/Cas9 system.

Primer	Primer sequence	Target region
**crRNAs for ATG5 knockout**		
crRNA-1	5′AAC UUG UUU CAC GCU AUA UC 3′	Exon 2
crRNA-2	5′AAG AGU AAG UUA UUU GAC GU 3	Exon 3
**crRNAs for MAP1LC3B knockout**		
crRNA-3	5′ CGG CGA CGA CGC GAG GGU CCG UUU UAG AGC UAU GCU 3′	Exon 2
crRNA-4	5′ AGA UCC CUG CAC CAU GCC GUG UUU UAG AGC UAU GCU 3′	Exon 2

Viruses and UV light inactivation. HSV-1 was wild-type F strain (GenBank accession number for the DNA genome sequence is GU734771). GHSV-UL46, obtained from the American Type Culture Collection (ATCC), is a recombinant HSV-1 labeled by fusing the green fluorescent protein (GFP) to the tegument structural protein VP11/12, the product of the UL46 gene (Willard, 2002). For UV inactivation (UV HSV-1), virions were exposed to 15 J/s UV light for 15 min. Inactivation was confirmed by virus titration, verifying the absence of viral replication.

Infection procedure and virus titration. Cells were infected at the appropriate m.o.i, and after 1 h of viral adsorption at 37°C, cells were rinsed and cultured in their respective growth medium. Viral titer was quantified in Vero cells by an endpoint dilution assay that determines the 50% tissue culture infectious dose (TCID50) using the Reed and Muench method (Ramakrishnan, 2016).

Antibodies. Primary antibodies were anti-ATG5 (D5F5U, Cell Signaling Technology), anti-ATG16L1 (A3637, ABclonal), anti-LC3B (2220SS, Sigma), anti-HSV-1 (B0114, Dako), anti-ICP0 (ab6513, abcam), anti-gE (ab6510, abcam), anti-ICP8 major DNA binding protein (ab20194, abcam), anti-β-actin-peroxidase antibody (A3854, Sigma). Horseradish peroxidase-conjugated secondary anti-IgG antibodies were purchased from Millipore (Billerica, MA, United States). Alexa-Fluor-488-conjugated were obtained from ThermoFisher Scientific (MA, United States).

Immunoblot analysis. Samples lysed in radioimmunoprecipitation assay buffer were mixed with Laemmli buffer and reduced with 2-mercaptoethanol (5% v/v). Then, the samples were subjected to SDS-PAGE in acrylamide gels and transferred to Immobilon-P PVDF membranes (Millipore). 15% acrylamide gels were required for the correct separation of LC3B-I/II. After blocking with 5% nonfat dry milk and 0.05% Tween 20 in PBS, blots were incubated overnight at 4°C with the corresponding antibody. The dilutions used for the antibodies were as follows: anti-ATG5 (1:1,000), anti-ATG16L1 (1:500), anti-LC3B (1:500), anti-HSV-1 (1:1,000), anti-ICP0 (1:1,000) and anti-ICP8 (1:500). After washing, the blots were incubated for 1 h at RT with a secondary antibody coupled to horseradish peroxidase. Anti-β-actin-peroxidase antibody (1:50,000) was directly incubated for 1 h at RT. Blots were revealed using an enhanced chemiluminescence Western blotting kit (ECL; Amersham, Little Chalfont, UK). The intensity of the bands was quantified using Fiji-ImageJ software. To correct for possible differences in well loading, the ratio between the protein of interest and the loading control (β-actin) was calculated. For the representation of the densitometry bar graphs, the calculated ratios were normalized to the value of the control sample (set to 1).

Immunofluorescence microscopy. Cell monolayers were grown on coverslips and, after HSV-1 infection, cells were fixed with 4% paraformaldehyde (PFA) in PBS for 15 min at RT. Cells were then permeabilized with 0.2% Triton X-100 in PBS and non-specific antibody binding was blocked with 3% BSA. The cells were then incubated for 1 h at RT with the primary antibodies diluted in PBS-BSA. After several washes, the cells were incubated with Alexa-Fluor-488 or -555 conjugated anti-mouse (1:500) diluted in PBS-BSA. Finally, DAPI was used to visualize the nuclei and coverslips were mounted with Mowiol. DAPI and Mowiol were purchased from Calbiochem (Merck Chemicals, Germany). Images were acquired using an LSM900 confocal laser scanning microscope coupled to an upright Axio Imager 2 microscope (Zeiss). Confocal images were processed using Fiji-ImageJ software.

Transient transfection of GFP-LC3 plasmid and quantification of LC3 puncta. The GFP-LC3 plasmid was generously provided by Dr. Natalia Reglero (CBMSO, Madrid, Spain). Cells were transfected with the plasmid using the X-tremeGENE 360 Transfection Reagent (Roche). The ratio of transfection reagent (μl) to DNA (mg) was 2:1. Cells were incubated with the reagent-DNA complex for 6 h. 24 h after transfection, cells were infected, or mock infected, with HSV-1. At 24 h post infection (hpi), the cells were fixed and labeled with anti-gE (1:1,000) to distinguish infected from mock cells. The deconvolution of confocal images was performed using the Huygens Professional software, in order to differentiate between large puncta and a cluster of adjacent small puncta. The quantification of GFP-LC3 puncta was performed using the “Analyze particles” option in Fiji-ImageJ software. The GFP-LC3 puncta per cell were quantified in at least 50 cells for each condition.

Reverse transcription quantitative PCR (RT-qPCR). Cells were cultured in 60-mm dishes and infected with HSV-1 at the appropriate m.o.i. Total RNA was extracted using the RNeasy Qiagene Mini Kit (Qiagene, Valencia, CA, United States). The EasyPure Viral DNA/RNA Kit (Transgen Biotech, ER201-01) was used for viral DNA/RNA extraction. Complete removal of genomic DNA from the RNA preparations was performed using the RapidOut DNA Removal Kit (ThermoFisher Scientific, K2981). RNA was quantified using a Nanodrop One spectrophotometer (Thermo Fisher Scientific). Reverse transcription reactions were performed using the iScript cDNA Synthesis Kit (Biorad PN170-8891) according to the manufacturer’s instructions. The thermal conditions consisted of the following steps: 5′ × 25°C; 20′ × 46°C and 1′ × 95°C.

qPCR reactions were performed on a CFX384 Real Time System C1000 thermal cycler (Bio-Rad). SsoFastTM EvaGreen Supermix (BIO-RAD) was used as the master mix. The thermal conditions consisted of the following steps: 30″ × 95°C + (5″ × 95°C and 5″ × 60°C) × 40 cycles. A melting curve from 60°C to 95°C (0.5°C/seg) was included at the end of the program to verify the specificity of the PCR. To evaluate the most suitable genes for normalization, the stability of different candidates –HMBS2, β-actin, PGK1, GAPDH, 18S rRNA and PP1α– was analyzed. The NormFinder algorithm suggested 18S rRNA (Table 2) as the most stable gene for normalization in HSV-1 infected cells. The primers used in this work (Table 2) were purchased from Integrated DNA Technologies (IDT).

**Table 2:** Primers used for qPCR analysis.

Primer	Sequence
18S ARNr (fwd)	5′ ATC CAT TGG AGG GCA AGT C 3′
18S ARNr (rev)	5′ GCT CCC AAG ATC CAA CTA CG 3′
UL30 (fwd)	5′ TGT TTC GCG TGT GGG ACA TA 3′
UL30 (rev)	5′ TTG TCC TTC AGG ACG GCT TC 3′
ICP8 (fwd)	5′ CAT GCC GGA TTT TAG CCG TG 3′
ICP8 (rev)	5′ AAA AAC GGA AGC GGG TAG GT 3′
ICP4 (fwd)	5′ CCT TTT TCC CAC CCA AGC ATC 3′
ICP4 (rev)	5′ CTG CTT GTT CTC CGA CGC C 3′
Us1 (fwd)	5′ GAG TTT GGG GAG TTT GAC 3′
Us1 (rev)	5′ GGC AGG CGG TGG AGA A 3′
UL47 (fwd)	5′ TGT TTT TCC GCC AAA CCC TG 3′
UL47 (rev)	5′ GGG GCT GGT TTT TGT TCG AC 3′

Forward (fwd), reverse (rev).

Flow cytometry. Cells were infected with the fluorescent GHSV-UL46 virus and harvested at various times post-infection. The cells were then fixed with 1% PFA and 1% FCS in PBS for 15 min at RT. Finally, cells were rinsed with PBS and GFP fluorescence was measured using a FACS Calibur flow cytometer (Becton Dickinson). Cytometry data were processed using FlowJo software.

Detection of HSV-1 binding and internalization. The amount of virus bound to and internalized by cells was measured as previously reported (Schneider et al., 2019; Wang et al., 2020; Song et al., 2022), with minor modifications. WT and ATG5 KO HOG cells were seeded in 60 mm dishes and differentiated for 24 h in DM. Cells were incubated for 1 h at 4°C, then infected with HSV-1 (m.o.i = 40) for another 1 h at 4°C. To determine virus binding, cells were rinsed three times with PBS 1X, and finally the DNA of viruses attached to the cell surface was extracted using the EasyPure Viral DNA/RNA kit (Transgen Biotech, ER201-01). To analyze viral entry, after the incubation with the virus at 4°C, cells were incubated for a further 1 h at 37°C to allow HSV-1 entry. Viral particles that bound to the cells but did not enter were then removed by washing three times with PBS (pH 3.0). Internalized viral DNA was extracted using the kit described above. Relative viral DNA levels were measured by qPCR using primers against the HSV-1 genes UL47 and Us1 (Table 2).

Statistical analysis. All statistical analysis were performed using GradPad Prism (version 8.0.1, GradPad Software, Inc.). Data were subjected to Mann–Whitney-U tests (non-parametric samples) to determine significant differences between groups. *p* values < 0.05 were considered statistically significant.

## 3 Results

### 3.1 HSV-1 inhibits the formation of autophagosomes in glial cell lines and OPCs

**Figure 1:**
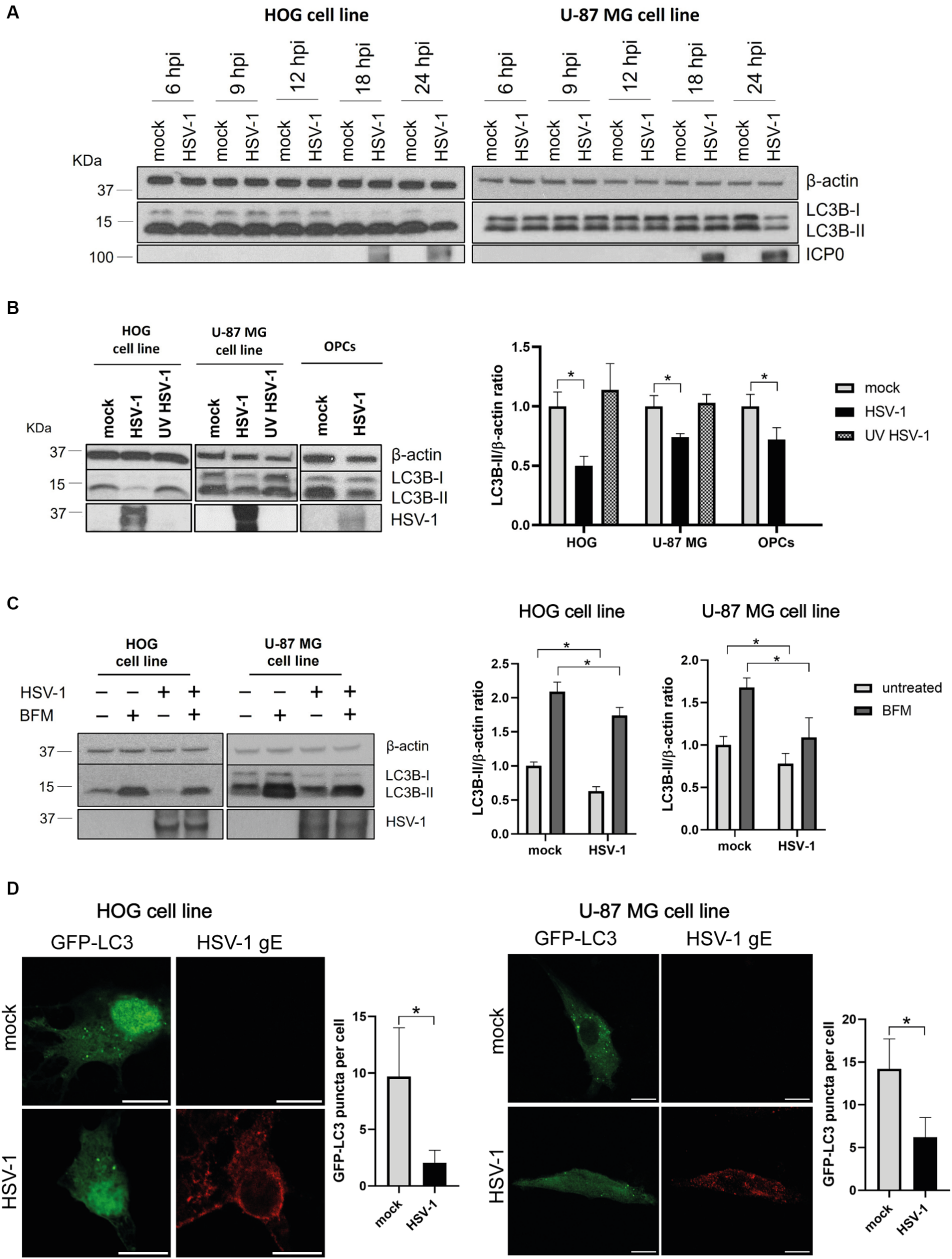
HSV-1 infection inhibits autophagosome formation in glial cells. **(A)** LC3 analysis by immunoblotting of HOG (m.o.i. = 1) and U-87 MG (m.o.i. = 5) cells infected for 6, 9, 12, 18, and 24 h. ICP0 was used as viral infection marker. **(B)** Representative WB for LC3B-I/II of cells infected with both non-inactivated and UV-inactivated HSV-1 for 24 h. OPCs were also infected with HSV-1 (m.o.i = 1) for 24 h. **(C)** Representative WB for LC3B-I/II of mock- and HSV-1 infected cells treated with 1 nM (HOG) or 10 nM BFM (U-87 MG) for 24 h. Untreated cells were used as control. Triplicate experiments were performed for each data point (*N* = 4). *p < 0.05. **(D)** Immunofluorescence images of both mock and HSV-1 infected cells transfected with GFP-LC3. The viral glycoprotein gE was used as a marker for viral infection. Scale bar, 10 μm. Triplicate experiments were performed and the GFP-LC3 puncta per cell were quantified in at least 50 cells for each condition (*p < 0.05).

To assess the impact of HSV-1 infection on glial autophagy, we used cell lines derived from a human oligodendroglioma (HOG cells) and a human astrocytoma (U-87 MG cells). These cells were infected with HSV-1 and the autophagic flux was monitored at different times post-infection by the autophagy marker LC3. Endogenous LC3 is detected as two bands following SDS-PAGE and immunoblotting: one corresponds to LC3-I, localized in the cytosol, and the other to LC3-II, which is conjugated with phosphatidylethanolamine (PE) and is present in autophagosomal membranes. At 24 hpi, infected cells exhibited decreased levels of LC3B-II compared to non-infected cells (Figure 1A). No changes in the amount of LC3B-II were observed up to 24 h (Figure 1A), suggesting that the reduced presence of autophagic membranes is significant only during the late stage of HSV-1 infection.

To determine whether fluctuations in autophagic flux are dependent or independent of viral gene expression, cells were infected for 24 h with both a non-inactivated and a UV light inactivated HSV-1, since UV light inactivation damages the viral genome and prevents HSV-1 replication. The ratio LC3B-II /β-actin was strongly diminished in infected HOG cells compared to the mock control. A significant, although less notable, reduction in the LC3B-II levels was also observed in infected U-87 MG cells (Figure 1B). No change in the levels of LC3B-II was noted in cells infected with UV-inactivated HSV-1, indicating that viral gene expression is required for autophagy modulation by HSV-1. We also conducted this experiment in OPCs infected for 24 h, and the results were analogous to those obtained with established cell lines (Figure 1B).

The autophagic flux is more accurately represented by comparing the amount of LC3-II between samples in the presence and absence of compounds that block autophagic degradation in a step downstream of autophagosome formation (Mizushima and Yoshimori, 2007). We used bafilomycin A1 (BFM), which is an inhibitor of the v-ATPase-dependent lysosomal acidification (Juhász, 2012). Cells were HSV-1 infected and treated with BFM with the aim of determining whether the decreased in LC3B-II levels during infection is due to a reduced autophagic activity or to an increased turnover in autophagosomes. LC3B-II levels of cells treated with BFM were diminished in infected cells compared to the mock-infected control (Figure 1C), which suggests that viral infection inhibits the formation of autophagosomes. However, basal autophagy in glial cells appears not to be entirely suppressed during the viral infection, as the LC3B-II form is still accumulated in infected cells.

Finally, we monitored autophagosomal membranes by LC3-immunofluorescence through transfection of a GFP-LC3 plasmid. The GFP-LC3 puncta per cell was significantly lower in HSV-1 infected cells than in the mock control (Figure 1D), confirming that infected glial cells have a lower amount of autophagosomal membranes.

### 3.2 Autophagy is not induced during the early stages of HSV-1 infection in glial cells

**Figure 2:**
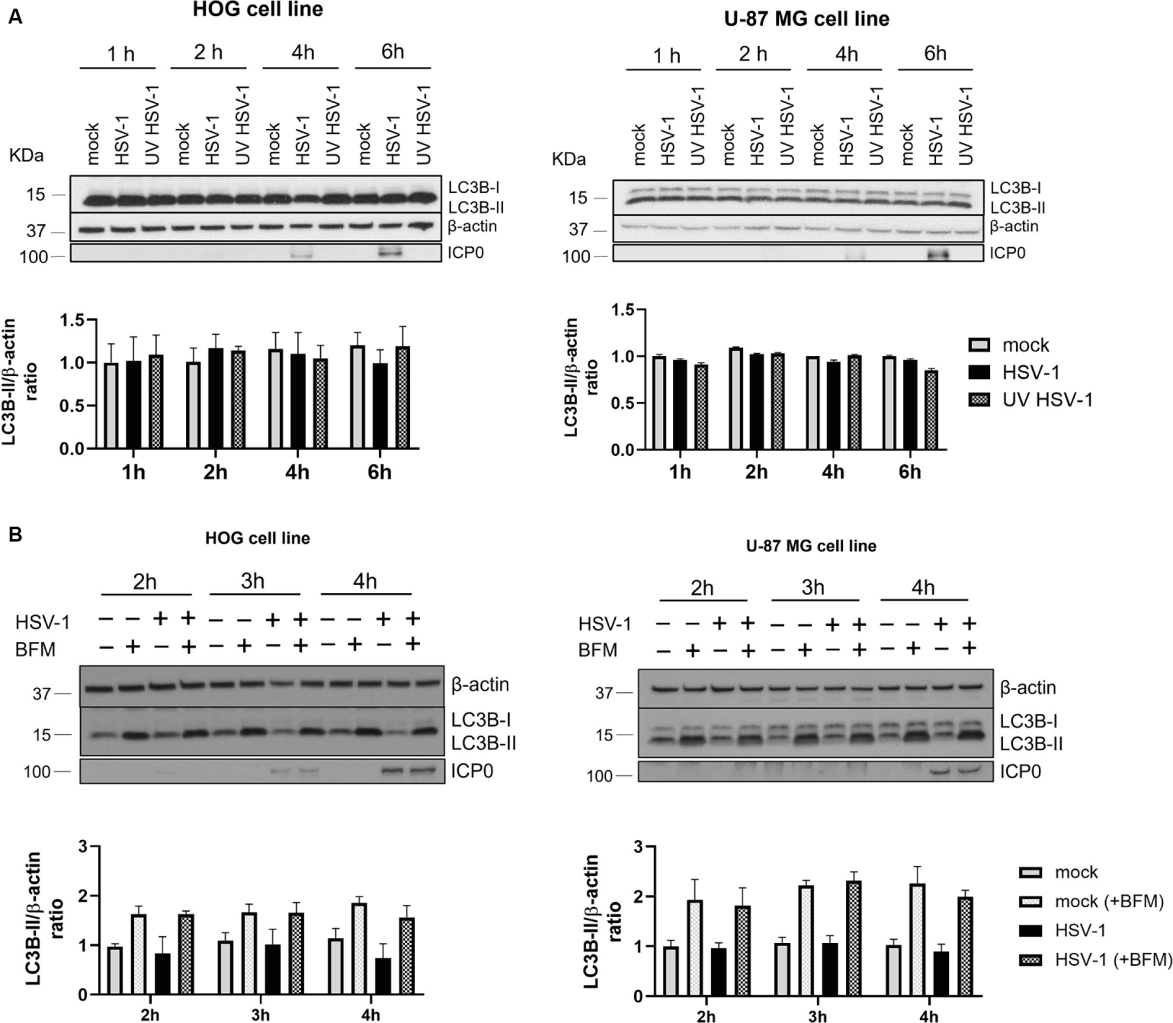
Autophagy is not induced in response to HSV-1 in glial cell lines. **(A)** HOG and U-87 MG cells were infected with both non-inactivated and UV-inactivated HSV-1 (m.o.i. = 40). Representative WB are shown for LC3B-I/II levels at 1, 2, 4, and 6 hpi. **(B)** Cells were non-treated or treated with 1 nM BFM (HOG cells) or 10 nM BFM (U-87-MG cells) for 3 h. Then, cells were infected with HSV-1, maintaining the BFM treatment during viral adsorption and infection. Mock-infected cells were used as control. Representative WB are shown for LC3B-I/II levels at 2, 3, and 4 hpi. Triplicate experiments were performed for each data point (*N* = 4). The comparison between the data of infected cells and the corresponding mock controls was not statistically significant.

Autophagy is a cellular defense mechanism, which is often stimulated in the context of a viral infection. To investigate if glial autophagy is induced in response to HSV-1, HOG and U-87 MG cells were infected at high m.o.i. with both a non-inactivated and a UV-inactivated HSV-1 and the LC3B-II/β-actin ratio was measured at early times post-infection. No differences in LC3B-II levels were observed between mock and infected cells (Figure 2A). We evaluated the LC3B-II/actin ratio in the presence and absence of BFM to confirm that glial autophagy was not induced in response to HSV-1, or that LC3B was being degraded in autolysosomes. The ratio of LC3B-II/β-actin remained unchanged in both mock and infected cells treated with BFM (Figure 2B). These results indicate that HSV-1 infection does not induce autophagy in glial cells. Thus, the later stage of HSV-1 infection does not inhibit induced-autophagy but leads to a decrease in the levels of basal autophagy.

### 3.3 Deletion of the ATG5 protein reduces HSV-1 infection

**Figure 3:**
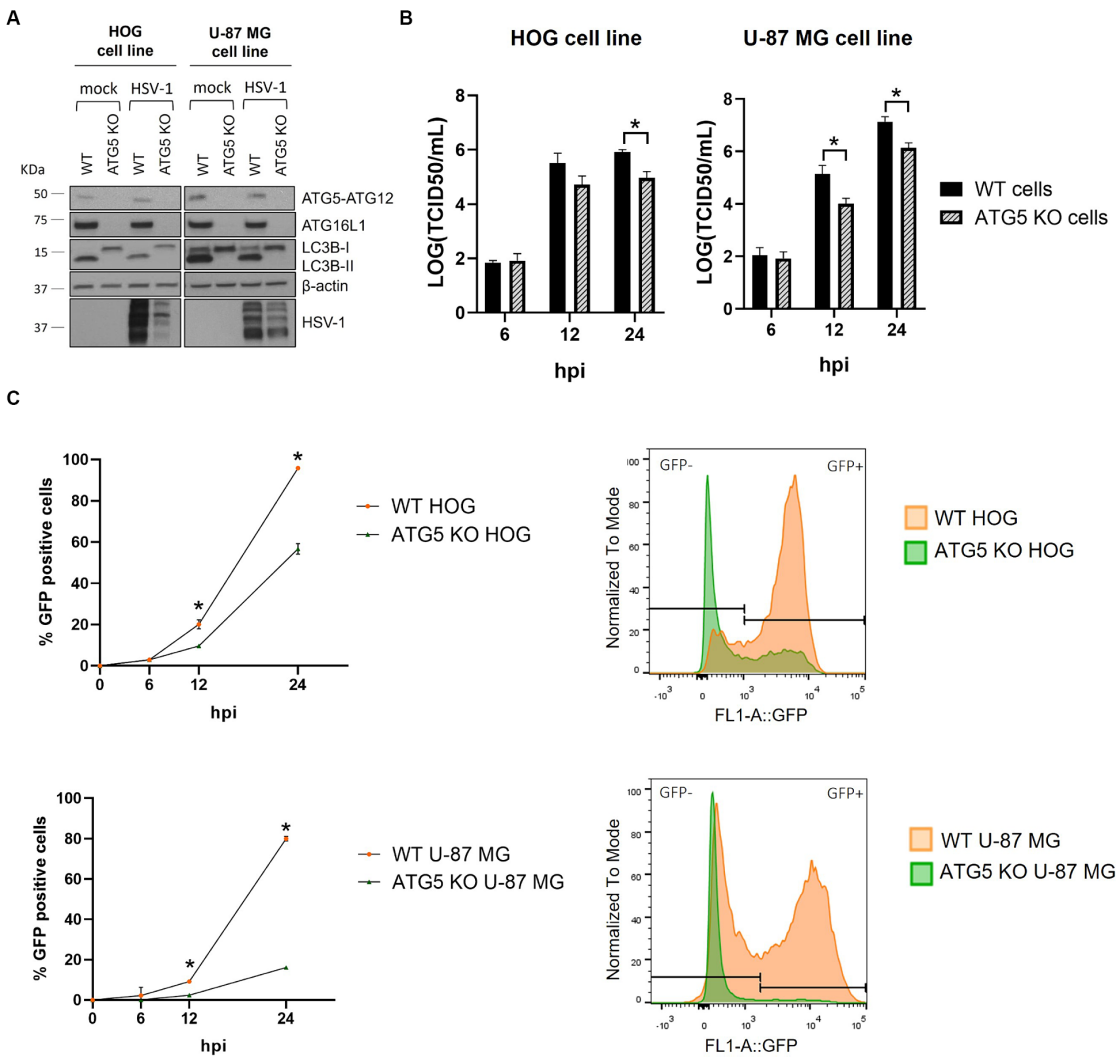
HSV-1 infection is significantly reduced in ATG5 KO glial cell lines. **(A)** The *ATG5* gene was knocked out by the CRISPR/Cas9 system. The lack of autophagy in KO cells was verified by checking the absence of ATG5-ATG12 complex and the PE-conjugation of LC3B-I by immunoblotting. **(B)** WT and ATG5 KO cells were infected with HVS-1 at a m.o.i of 1 (HOG) and at a m.o.i of 5 (U-87 MG). Then, cells were harvested at 6, 12, and 24 h to determine the TCID50/mL. **(C)** WT and ATG5 KO cells were infected with GHSV-UL46 and the percentage of infected cells was measured by flow cytometry. Plots represent the histograms of GFP-positive and GFP-negative cells at 24 hpi (*N* = 4, *p < 0.05).

To analyze the impact of autophagy on HSV-1 infection of glial cells, we knocked out the *ATG5* gene in the HOG and U-87 MG cell lines using the CRISPR/Cas9 system. The ATG5 protein is covalently conjugated to ATG12 and interacts with ATG16L1 to form the ATG12-ATG5/ATG16L1 complex. The absence of the ATG5-ATG12 complex in knockout (KO) cells was confirmed by immunoblotting (Figure 3A). As it was previously described (Fujita et al., 2009), ATG16L1 is almost undetectable in cells lacking ATG5 (Figure 3A). One proposed explanation is that these proteins need to be assembled into the ATG12-ATG5/ATG16L1 complex to be stabilized, and the absence of one of them reduces the half-life of the others (Hamaoui and Subtil, 2022). The ATG12-ATG5/ATG16L1 complex catalyzes the conjugation of the cytosolic protein LC3B-I to PE, resulting in the lipidated form LC3B-II, which is essential for the formation of autophagosomes. The lack of LC3B-II and the accumulation of LC3B-I in ATG5 KO cells confirmed the successful blockage of autophagy (Figure 3A). Finally, we analyzed the expression of the ATG5-12-16 L1 complex and the lipidation of LC3B during HSV-1 infection of wild-type (WT) and ATG5 KO cells (Figure 3A). The levels of ATG5-12-16 L1 remained unchanged between mock and infected cells. LC3B-II levels were reduced in HSV-1 infected cells, as previously observed (Figure 1B). Infected ATG5 KO cells still showed no expression of ATG16L1 and LC3B-II. Interestingly, a significant reduction in HSV-1 infection was observed in ATG5 KO cells (Figure 3A).

To evaluate the impact of ATG5 deletion on HSV-1 infection, we monitored the progression of the infection in WT and ATG5 KO cells by viral production titration (Figure 3B) and flow cytometry (Figure 3C). Interestingly, HSV-1 infection in ATG5 KO cells was significantly decreased at 12 hpi, with the reduction being more pronounced at 24 hpi. The impairment of infection was particularly notable in the U-87 MG cell line, where the percentage of infected cells was five times lower in ATG5 KO cells.

### 3.4 ATG5 plays a proviral non-autophagic role in HSV-1 infection

**Figure 4:**
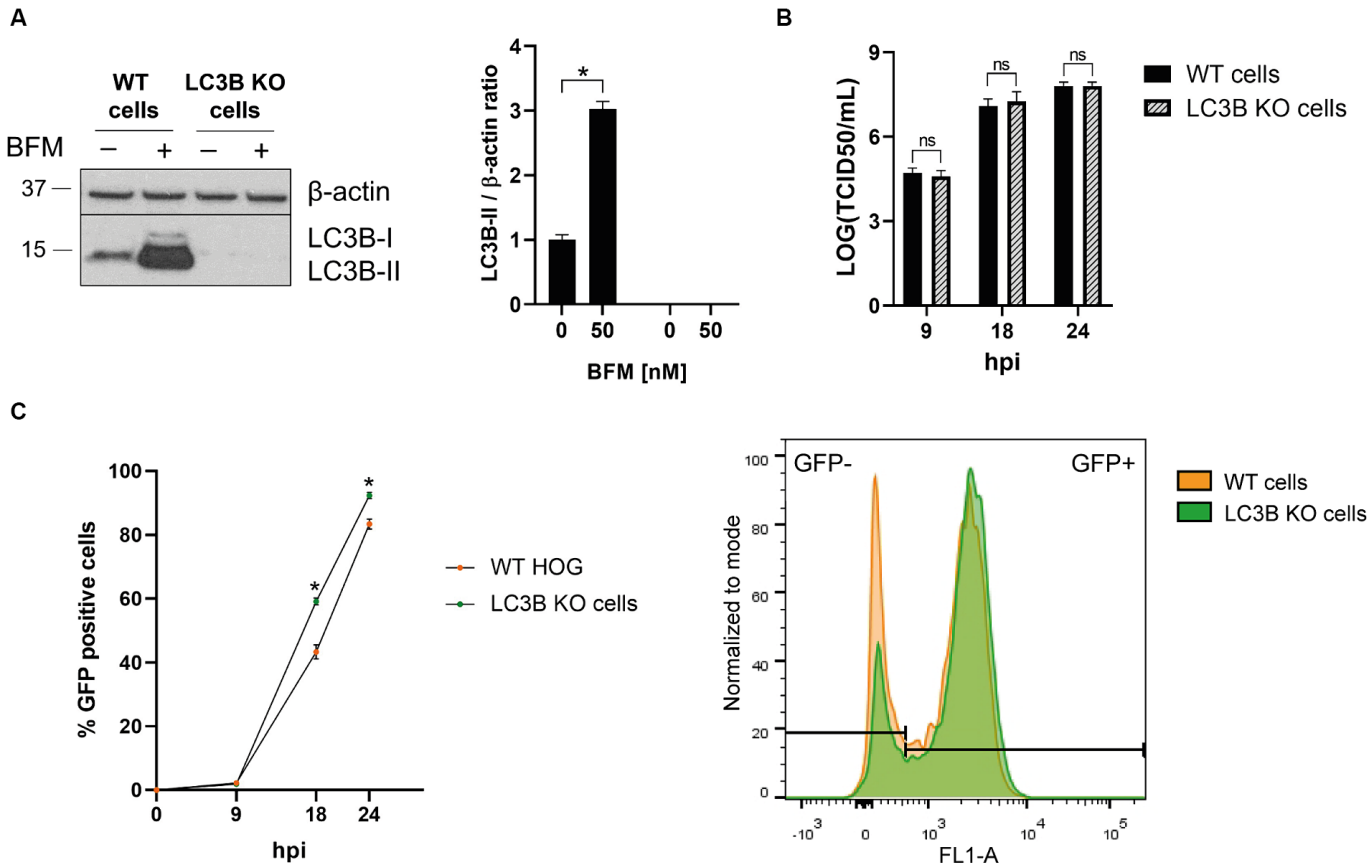
The absence of autophagy has almost no effect on HSV-1 infection in glial cells. **(A)** The *MAP1LC3B* gene was knocked out in the HOG cell line by the CRISPR/Cas9 system. WT and KO cells were untreated and treated with 5 nM BFM for 24 h, and the absence of LC3B in KO cells was verified by immunoblot analysis. **(B)** WT and LC3B KO cells were HSV-1 infected (m.o.i = 1) and harvested at 9, 18, and 24 h to determine the TCID50/mL. **(C)** WT and LC3B KO cells were infected with GHSV-UL46 and the percentage of infected cells was measured by flow cytometry. Plots represent the histograms of GFP-positive and GFP-negative cells at 24 hpi (*N* = 4, *p < 0.05).

ATG5 is essential for the formation of autophagosomes (Hu and Reggiori, 2022), but it has also non-canonical functions unrelated to autophagy that are largely unknown and have been proposed to influence viral infections (Guévin et al., 2010). To determine whether the decrease in HSV-1 infection in ATG5 KO cells is due to the absence of ATG5-mediated autophagy or a consequence of a non-canonical role of ATG5, we knocked out the *MAP1LC3B* gene. The absence of LC3B was confirmed by immunoblotting. LC3B was not detected in LC3B KO cells, even after the treatment with BFM (Figure 4A). The titration of viral production did not reveal any significant differences between the infection of WT and LC3B KO cells (Figure 4B). The percentage of infected cells was slightly higher in LC3B KO cells compared to WT cells at later stages of infection (Figure 4C). The findings suggest that basal autophagy does not have a significant antiviral effect on HSV-1 infection in glial cells. Therefore, the observed decrease in infection in ATG5 KO cells is likely due to a non-canonical role of ATG5.

### 3.5 HSV-1 DNA transcription and replication are impaired in the absence of ATG5

**Figure 5:**
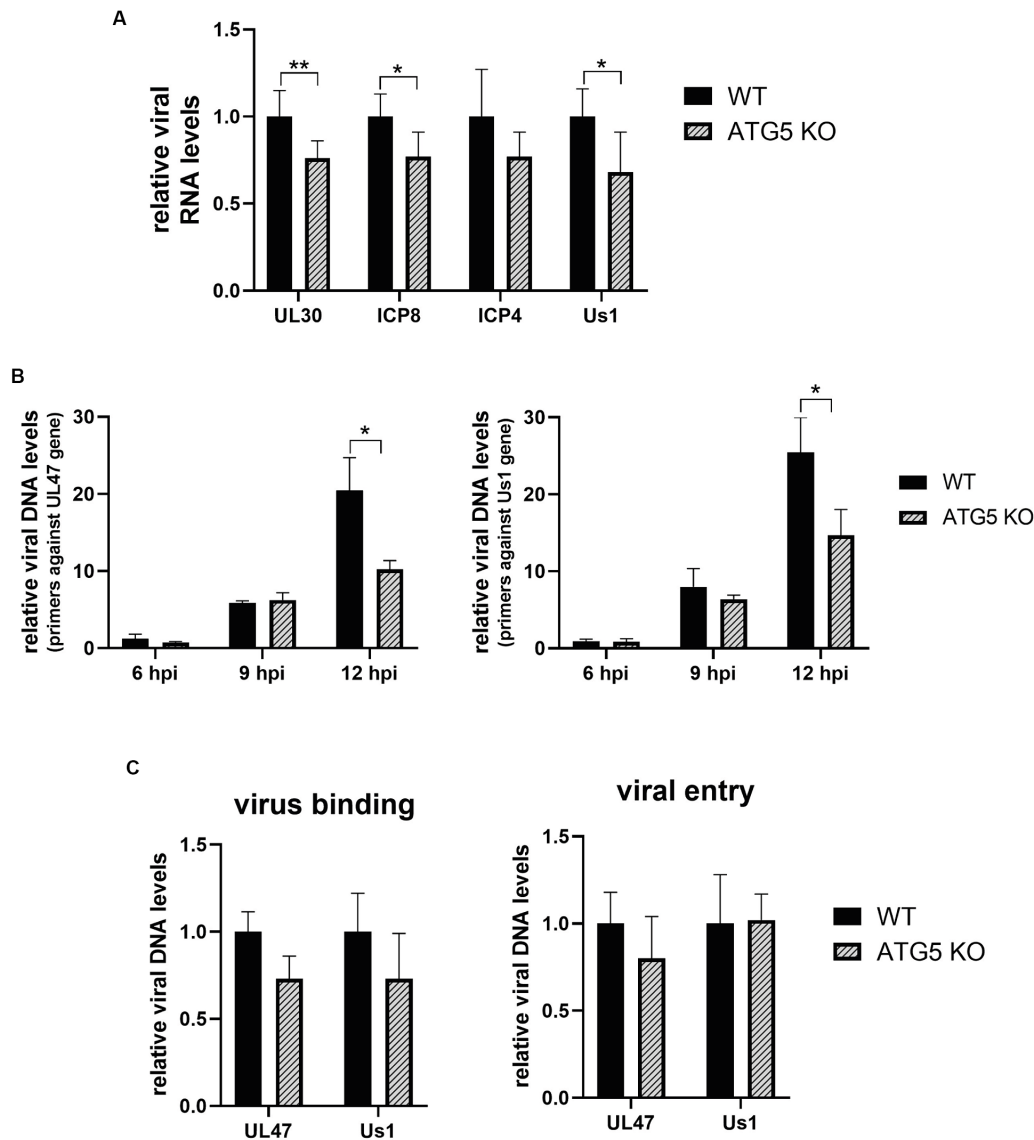
HSV-1 DNA transcription and replication are decreased in the absence of ATG5, but no changes in virus binding or entry are detected. **(A)** ATG5 KO and WT HOG cells were infected with HSV-1 (m.o.i = 40) and the relative levels of viral RNA was determined by RT-qPCR at 4 hpi. **(B)** ATG5 KO and WT HOG cells were infected with HSV-1 (m.o.i = 20) and the relative levels of viral DNA were determined by qPCR at 6, 9, and 12 hpi. **(C)** Prechilled ATG5 KO and WT HOG cells were incubated for 1 h at 4°C with HSV-1 (m.o.i = 40). To analyze virus binding, total DNA of bound viruses was measured by qPCR. To determine viral entry, cells were incubated with the virus for an additional hour at 37°C to allow HSV-1 entry. The DNA from internalized viruses was then extracted and quantified by qPCR (*N* = 6, *p < 0.05, **p < 0.01).

As HSV-1 infection was significantly impaired at 12 hpi, we sought to investigate the potential impact of ATG5 deletion on the initial stages of infection, starting by analyzing the transcription and/or replication of the viral genome. To determine whether viral transcription was affected by the absence of ATG5, WT and ATG5 KO cells were HSV-1 infected, and the viral DNA/RNA was extracted at 4 hpi. The relative RNA levels of the immediate-early gene US1 and the early genes UL30 and ICP8 were significantly lower in ATG5 KO cells compared to WT cells. No significant differences in the expression of the immediate-early gene ICP4 were observed (Figure 5A). The product of the US1 gene, the ICP22 protein, promotes the elongation of viral transcription, whereas the DNA polymerase (UL30) and the single-stranded DNA binding protein (ICP8) are involved in viral DNA replication (Packard and Dembowski, 2021). Therefore, reduced transcription of these viral genes may affect HSV-1 genome replication. To analyze whether HSV-1 DNA replication, viral DNA/RNA was extracted from infected KO and WT cells at 6, 9, and 12 hpi. The relative levels of viral DNA were determined by qPCR using primers against two viral genes (UL47 and US1). A significant reduction in viral replication was observed in ATG5 KO cells at 12 hpi (Figure 5B).

The reduction in viral transcription and replication could be a consequence of an ineffective binding and/or entry of the virus into the KO cells. To rule out this possibility, we first examined the amount of HSV-1 bound to the cells by quantifying the viral genome. For that, prechilled ATG5 KO and WT cells were infected with HSV-1 for 1 h at 4°C. Finally, HSV-1 DNA from cell-attached viruses was extracted and quantified by qPCR. To determine viral entry, after 1 h of viral adsorption at 4°C, cells were incubated for an additional hour at 37°C to allow viral entry. Cells were then rinsed with PBS pH 3.0 to remove any virus remaining on the cell surface, and internalized viruses were detected by qPCR. No significant differences in virus binding and entry were observed between ATG5 KO and WT cells (Figure 5C).

### 3.6 Formation of HSV-1 replication compartments is delayed in the absence of ATG5

ATG5 (Guévin et al., 2010) and ATG16L1 (Hwang et al., 2012) have been proposed to interact with viral replication complexes. We investigated whether reduced viral DNA replication in the absence of ATG5 could be related to a defective formation of HSV-1 replication compartments (RCs). The formation of these compartments goes through four stages, which can be distinguished by analyzing the viral protein ICP8 by immunofluorescence. Diffuse nuclear staining of ICP8 is observed during the stage II, which is followed by a discrete punctate ICP8 foci. The punctate foci correspond to HSV-1 prereplicative sites, which characterize the stage III. The coalescence of these small prereplicative sites leads to the formation of larger punctate foci to, finally, generate large globular structures corresponding to the mature RCs (stage IV) (Livingston et al., 2008).

**Figure 6:**
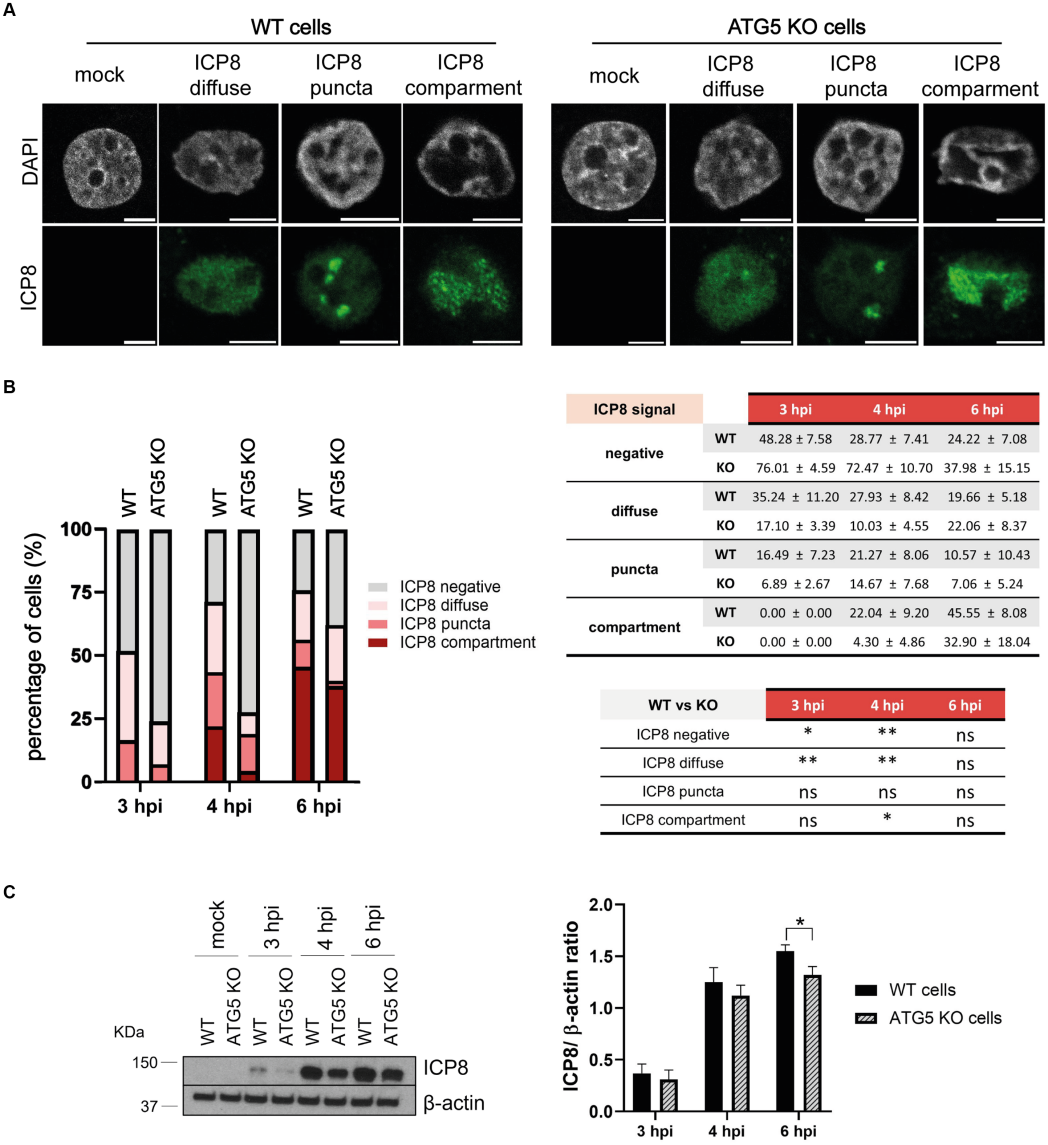
The absence of ATG5 leads to a delay in the formation of HSV-1 replication compartments. **(A)** WT and ATG5 KO HOG cells were infected with HSV-1 (m.o.i = 40) and fixed at 3, 4, and 6 hpi. Formation of ICP8 foci was assessed by immunofluorescence. **(B)** The relative abundance of the different stages of replication compartments was quantified by counting 300 infected cells and categorizing them based on ICP8 signal as negative, diffuse (stage II), puncta (stages III-IV), or compartment (stage IV). **(C)** Representative WB and relative densitometric bar graphs of ICP8 (*N* = 4, *p < 0.05, **p < 0.01).

The formation of HSV-1 RCs was monitored in both ATG5 KO and WT cells by immunofluorescence analysis (Figure 6A). A delay in the formation of RCs was detected in ATG5 KO cells at 3 and 4 hpi, which corresponds to the initial stages of formation of HSV-1 RCs (Figure 6B). The amount of ICP8 was also measured by immunoblotting at 3, 4 and 6 hpi. Lower levels of ICP8 were detected in infected ATG5 KO cells compared to WT cells, confirming previous results (Figure 6C).

## 4 Discussion

Understanding the relationship between autophagy and HSV-1 infection is becoming increasingly difficult. One of the major challenges is the wide variation of results between cell types (Ripa et al., 2022). HSV-1 has been proposed to inhibit the early autophagosome formation in primary cultures of neurons and fibroblasts (Tallóczy et al., 2006; Orvedahl et al., 2007). In contrast to primary neurons, in neuroblastoma cells HSV-1 interferes with late autophagosomal stages, causing an incomplete autophagy response (Santana et al., 2012a). Subsequent studies indicate that HSV-1 also counteracts late autophagosomal maturation in dendritic cells (Leib and Gobeil, 2012; Budida et al., 2017). The analysis of autophagy in other cell types, including corneal epithelial and retinal ganglion cells, did not reveal any changes in autophagic flux during infection (Yakoub and Shukla, 2015b). The present work examines the impact of HSV-1 on autophagy in glial cells. Our results demonstrate that HSV-1 inhibits the formation of autophagosomes in human oligodendroglioma- and astrocytoma-derived cell lines during the late stage of infection. Similar findings were uncovered in primary cultures of mouse OPCs. However, while neuronal autophagy is induced in the early stages of HSV-1 infection through the IFN-inducible PKR signaling pathway (Tallóczy et al., 2006), glial autophagy was not stimulated in response to HSV-1. Therefore, while HSV-1 leads to a reduction of induced-autophagy in neurons, in glial cells the virus directly decreases the levels of basal autophagy.

The inhibition by HSV-1 of a critical pathway such as autophagy, which is essential for the survival and maturation of glial cells, may have implications for the development and progression of demyelinating diseases like MS (Liang and Le, 2015). MS is the most common inflammatory autoimmune disease of the CNS, thus leading to demyelinated axons vulnerable to injury and degeneration. Partial restoration of lost OLs and the (re)establishment of myelin sheaths around demyelinated axons can take place through the activation, recruitment, and differentiation of OPCs (Zhao and Jacob, 2023). However, the remyelination of MS lesions often fails (Gruchot et al., 2019). Both the efficacy and the rate of OPC recruitment are critical determinants of remyelination. In certain cases, non-remyelinating lesions are abundant in OPCs and immature OLs, but there is a failure in transition into remyelinating OLs (Lucchinetti et al., 1999; Nsi et al., 2002; Kuhlmann et al., 2008). The reason behind remyelination failure in MS remains unclear. Furthermore, it appears that multiple cell types beyond just myelinating cells may be implicated in this process. Astrocytes also play a crucial role in the repair of MS lesions, promoting the recruitment of microglia to clear myelin debris (Skripuletz et al., 2013) and the proliferation of OPCs (Lentferink et al., 2018). Further research is necessary to ascertain whether the potential inhibition of glial autophagy by HSV-1 infection could be involved in the failure of remyelination in MS lesions.

Finally, this study investigates the impact of autophagy on HSV-1 infection of glial cells. The absence of LC3B, and therefore autophagy, did not appear to affect viral infection. These findings suggest that autophagy may not be a relevant antiviral mechanism in glia, unlike neuronal autophagy (Yordy et al., 2012). However, the absence of the autophagy-related protein ATG5 led to a significant reduction in HSV-1 infection. The role of ATG5 in glial cells during HSV-1 infection is not well understood, despite its importance in OL myelination (Bankston et al., 2019) and astrocyte differentiation (Wang et al., 2014). Altered ATG5 expression can dysregulate both immune and nervous system cells, which has been proposed to influence demyelination and neurodegeneration in MS (Baeva and Camara-Lemarroy, 2023). In the context of a viral infection, ATG5 has been widely considered an antiviral mechanism due to its participation in autophagy (Tallóczy et al., 2006), although the precise role of this protein in HSV-1 infection is still under discussion. Results consistent with ours have been obtained in experiments with HSV-2, in which Atg5^−/−^ MEFs shown a dramatic decrease in HSV-1 infection. The authors proposed that a minimum level of basal autophagy was required for the success of HSV infection (Yakoub and Shukla, 2015a). However, we verified that the absence of basal autophagy did not affect HSV-1 infection in glial cells. Recent research shows that autophagy-related proteins (ATGs) participate in numerous non-autophagic signaling processes (Subramani and Malhotra, 2013). ATG5 participates not only in the canonical and non-canonical autophagy, but also in regulating immune responses, apoptosis (Ye et al., 2018), and DNA damage (Maskey et al., 2013; Simon and Friis, 2014; Sun et al., 2021). Regarding viral infections, the non-canonical functions of ATG5 can be exploited to enhance viral replication. For instance, ATG5 can facilitate the initiation of hepatitis C virus infection by interacting with the RNA polymerase (Guévin et al., 2010). Additionally, it seems that the ATG12–ATG5 conjugate can hinder innate antiviral immune responses against the vesicular stomatitis virus (Jounai et al., 2007; Takeshita et al., 2008). Furthermore, ATG5 may have a significant function as a central mediator of a non-canonical autophagy pathway used by HIV-1 to hinder host responses (Judith and Berlioz-Torrent, 2023). On the other hand, ATG5 can also obstruct viral infections by playing a pivotal, non-degradative role in IFNγ-mediated antiviral defenses (Hwang et al., 2012).

Hence, a potential unexplored function of ATG5 may be affecting the viral cycle of HSV-1. In the present study, we have reported that the transcription and replication of HSV-1 genes were significantly impaired in the absence of ATG5. Moreover, the initiation of the formation of HSV-1 RCs was considerably delayed in cells that lack ATG5, indicating a possible involvement of this protein in the formation of HSV-1 RCs. However, further experiments are necessary to determine the mechanism of action of ATG5 and whether the delay in the initial phases of RCs formation directly causes the decrease in viral DNA replication or ATG5 is involved in other aspects of viral replication. In addition, it is suggested that ATG5 may have different roles depending on its cellular location (Simon and Friis, 2014). Further experimentation would be required to ascertain whether ATG5 plays a distinct role in the HSV-1 cycle contingent upon its nuclear or cytosolic distribution. Finally, it is also important to note that the CRISPR technology has experimental limitations and, it cannot be ruled out that the reduction in HSV-1 infection may be due to off-target effects of the knock-out cells.

In summary, the presented results introduce a new concept of autophagy in herpesvirus infections. It is proposed that HSV-1 inhibits the autophagic flux in glial cells not to avoid xenophagy and immune responses, but to exploit certain proteins of the autophagic machinery, such as ATG5, which possess non-autophagic roles that may aid in promoting the viral cycle.

## 5 Conclusion

Dysregulation of autophagy in glial cells has been linked to demyelinating diseases. Our findings demonstrate that basal autophagy of glial cells is impaired during HSV-1 infection, which emphasizes the importance of studying the impact of HSV-1 on demyelinating processes. Interestingly, although HSV-1 inhibits autophagy, the virus requires ATG5 for the success of viral transcription and replication, which reveals the complexity and multifunctionality of the autophagic machinery components. Our hypothesis is that HSV-1 inhibits the autophagic flux in glial cells to take advantage of the non-autophagic roles of certain components of the autophagic machinery. However, further research is needed to clarify the complex relationship between autophagy and HSV-1 in the CNS and the implication that this interplay has on neurodegeneration.

## Data availability statement

The original contributions presented in the study are included in the article/supplementary material, further inquiries can be directed to the corresponding author.

## Ethics statement

The animal study was approved by Ethical Review Board of Consejo Superior de Investigaciones Biológicas-CSIC and Comunidad de Madrid. The study was conducted in accordance with the local legislation and institutional requirements.

## Author contributions

IR: Conceptualization, Formal analysis, Investigation, Writing – original draft. SA: Writing – review & editing. FJ-P: Methodology, Writing – review & editing. BF: Methodology, Writing – review & editing. FC: Funding acquisition, Methodology, Writing – review & editing. MA: Writing – review & editing. RB-M: Conceptualization, Funding acquisition, Project administration, Supervision, Writing – review & editing. JL-G: Conceptualization, Funding acquisition, Project administration, Supervision, Writing – review & editing.
